# Equity in the Developmental Dysplasia of the Hip (DDH) Diagnosis and Treatment: A Retrospective Cohort Study to Unravel the Effect of Area Deprivation and the Insurance Type

**DOI:** 10.7759/cureus.56956

**Published:** 2024-03-26

**Authors:** Samantha L Ferraro, Sarah Dance, Delara Rajabi, Ahmed Elabd, Sean Tabaie

**Affiliations:** 1 Orthopaedic Surgery, George Washington University School of Medicine and Health Sciences, Washington DC, USA; 2 Orthopaedic Surgery, Children’s National Hospital, Washington DC, USA; 3 Orthopaedic Surgery, Children's National Hospital, Washington DC, USA

**Keywords:** pavlik harness, socioeconomic factors, area deprivation index, pediatric orthopedic surgery, developmental dysplasia of the hip

## Abstract

Background

Timely diagnosis of developmental dysplasia of the hip (DDH) is crucial for implementing less invasive treatment. However, socioeconomic barriers may lead to late diagnoses. The Area Deprivation Index (ADI) is an indicator of the socioeconomic challenges experienced by patients and their families. The primary objective is to investigate if the age at which DDH is diagnosed and the treatment protocol are influenced by the ADI or the insurance type.

Materials and methods

Using International Classification of Diseases-Tenth Edition (ICD-10) codes, newly diagnosed DDH patients (age under 10 years) from 2020 to 2023 were retrospectively identified at our pediatric tertiary center. Patients were categorized into four groups based on ADI percentile: (1) 1-10th percentile, (2) 11-20th percentile, (3) 21-40th percentile, and (4) 41-100th percentile. They were also stratified by insurance type. Age at diagnosis and treatment protocol (non-operative vs. operative) were collected and compared between the different ADI groups and insurance groups. Operative treatment was defined as open reduction with or without femoral/pelvic osteotomy.

Results

A total of 327 patients satisfied the inclusion criteria and had available ADI scores for analysis. The average age at diagnosis was notably lower in ADI group 1 compared to all other ADI groups (p < 0.05) and considerably lower for patients with commercial insurance compared to those with public (p = 0.0002). The rate of surgical treatment was markedly lower in ADI group 1 compared to ADI groups 2 and 3 (both p < 0.05) and notably lower for those with commercial insurance compared to public (p = 0.0005). ADI groups 2-4 showed no significant differences in average age at diagnosis or surgical treatment rate.

Conclusion

The study demonstrates that socioeconomic factors affect the diagnosis and, consequently, the treatment course of DDH. Specifically, patients residing in areas with lower levels of deprivation tend to be diagnosed at a younger age and undergo surgical treatment less frequently.

## Introduction

Developmental dysplasia of the hip (DDH) is the leading cause of early-onset degenerative hip arthritis, with an incidence of one in 100 to 1000 live births [1-3]. Timely identification and intervention are crucial for achieving properly functioning and pain-free hip joints throughout adulthood. The chance of successful non-operative treatment, including Pavlik harness and closed reduction, drops as the soft tissue contracts, hindering the success of non-operative treatment. However, with proper screening and timely diagnosis, the hip can often be treated without surgical intervention. Consequently, identifying DDH at an earlier age is associated with better functional outcomes and lower rates of surgical treatment [4,5].

It is well documented in the literature that many disparities exist in pediatric medical and dental care among different racial/ethnic backgrounds [6]. Patients’ demographics, socioeconomic factors, and insurance type can impact a patient’s ability to receive adequate healthcare on time [7,8]. While several factors have been identified that influence early screening and treatment for the DDH population, the existing body of literature has limitations in terms of precisely identifying the role socioeconomic status has in early diagnosis and treatment [7]. 

The Area Deprivation Index (ADI) provides a percentile-based scoring system based on residential addresses, encompassing factors such as income, education, employment, and housing quality in a given area [9]. A higher ADI percentile equates to a neighborhood having a higher number of socioeconomic disparities. In this study, we employed the ADI as an indicator of the socioeconomic challenges faced by patients and their families. The primary objective of the study was to investigate whether the age at which DDH was diagnosed and the treatment protocol were influenced by the ADI or the insurance type.

## Materials and methods

The study was an IRB-approved, retrospective chart review of patients diagnosed with DDH at our tertiary pediatric care facility within a period spanning from January 1, 2020, to December 31, 2023. Patients who were 10 years old or younger at the time of their DDH diagnosis were identified using International Classification of Diseases-Tenth Edition (ICD-10) codes Q65.0-Q65.6. All patients at our institution who were 10 years old or younger at the time of diagnosis and had a diagnosis of hip dysplasia, home address zip code, insurance information, and treatment protocol in their medical charts were included in the study. Patients older than 10 years old at the time of DDH diagnosis or who had incomplete chart data were excluded. An initial institutional search yielded a total of 330 patients by ICD-10 codes and age restriction. Three patients were further excluded from the study due to incomplete chart data. 

To identify the socioeconomic status of each patient, the zip code of their home address was employed to derive an ADI score, represented as a national percentile ranging from 1 to 100. The lower the percentile, the higher the socioeconomic status of that neighborhood. For example, a percentile of 1 equates to having the lowest level of disadvantage within the United States, whereas a percentile of 100 equates to the highest level of disadvantage [10]. For the purpose of this study, patients were stratified into four distinct groups according to their ADI score: group 1 represented any ADI scores from the 1st to 10th percentile, group 2 represented ADI from the 11th to 20th percentile, group 3 represented ADI from the 21st to 40th percentile, and group 4 represented ADI from the 41st to 100th percentile. Insurance type data (commercial insurance vs. public insurance) was also documented.

Treatment protocol data were collected from the electronic medical records for each patient, including the type of treatment (operative vs. non-operative). The operative group consisted of patients who underwent an open reduction, osteotomy (pelvic or femoral), and/or adductor tenotomy, while the remaining patients were in the non-operative group (bracing, arthrogram, or closed reduction under anesthesia). For each patient treated operatively, the operative reports were reviewed to subsequently categorize the operative patients into two groups: osteotomy (pelvic or femoral) and non-osteotomy (open reduction or adductor tenotomy). To comprehensively assess the findings, the study compared the average age at which DDH was diagnosed and the frequency of surgical treatment across the different ADI groups as well as the two insurance groups.

Statistical analysis

The age assessment at diagnosis in different ADI and insurance-type groups was performed using a T-test to determine differences in means. The assessment of the rate of surgical treatment between different ADI and insurance-type groups was performed using a Z-test for differences in proportions. The ADI groups 2-4 were each compared to group 1 (most advantaged) to yield the p-value. A p-value less than 0.05 was considered statistically significant.

## Results

In total, 330 patients aged 10 or younger with a new DDH diagnosis from 2020 to 2023 were identified using ICD-10 codes. Three patients were excluded due to a lack of available ADI data, leaving 327 patients available for analysis.

Outcome 1: age at DDH diagnosis

The average age at diagnosis of DDH among the entire cohort was 3.46 years (SD, 3.08). The average age at diagnosis for group 1 (most advantaged) was significantly younger than in group 2 (2.62 years vs. 3.95 years, p = 0.0029), group 3 (2.62 years vs. 3.72 years, p = 0.0111), and group 4 (2.62 years vs. 3.65 years, p = 0.0481) (Table 1). The average ages at diagnosis for groups 2-4 did not differ significantly from each other (all p > 0.05). The average age at diagnosis for commercially insured patients was significantly younger than for publicly insured patients (2.70 years vs. 3.97 years, p = 0.0002) (Table 2).

**Table 1:** Average age at DDH diagnosis based on ADI score DDH = developmental dysplasia of the hip; ADI = Area Deprivation Index; n = number; SD = standard deviation

	ADI national percentile range	Patients (n)	Mean age at diagnosis		p-value (vs. group 1)
			Days (SD)	Years (SD)	
Group 1	1-10th	92	957.78 (1001.29)	2.62 (2.74)	-
Group 2	11-20th	79	1440.66 (1087.07)	3.95 (2.98)	0.0029
Group 3	21-40th	103	1358.97 (1163.86)	3.72 (3.19)	0.0111
Group 4	41-100th	53	1332.21 (1228.26)	3.65 (3.37)	0.0481

**Table 2:** Average age at DDH diagnosis based on insurance type Public = Medicaid, Tricare, and Embassy insurance coverage; other = self-pay or grants; n = number; SD = standard deviation; DDH = developmental dysplasia of the hip; ADI = Area Deprivation Index

Insurance type	# of patients (n)	Mean age at diagnosis		p-value (commercial vs. public)
		Days (SD)	Years (SD)	
Commercial	138	987.04 (1112.58)	2.70 (3.05)	0.0002
Public	183	1448.42 (1084.50)	3.97 (2.97)	-
Other	6	1872.83 (1387.00)	-	-

Outcome 2: rate of surgical DDH treatment

The average rate of surgical treatment for group 1 (most advantaged) was significantly lower than in group 2 (16% vs. 33%, p = 0.0111) and group 3 (16% vs. 31%, p = 0.0160) but not in group 4 (16% vs. 28%, p = 0.0854) (Table 3). The surgery rates for groups 2-4 did not differ significantly from each other (all p > 0.05). Of the surgeries performed, group 4 had a greater proportion of osteotomies (93%) compared to groups 2 (81%) and 3 (72%), which had a greater proportion of osteotomies compared to group 1 (67%) (Table 3). No statistical analysis was performed with this sub-group analysis due to the small sample sizes.

**Table 3:** Rate of surgical treatment based on ADI ADI = Area Deprivation Index

	ADI national percentile range	Total surgery		Surgery subtypes	
		%	p-value (vs. group 1)	Osteotomy (%)	Non-osteotomy (%)
Group 1	1-10th	16% (15/92)	-	67% (10/15)	33% (5/15)
Group 2	11-20th	33% (26/79)	0.0111	81% (21/26)	19% (5/26)
Group 3	21-40th	31% (32/103)	0.0160	72% (23/32)	28% (9/32)
Group 4	41-100th	28% (15/53)	0.0854	93% (14/15)	7% (1/15)

The average rate of surgical treatment for commercially insured patients was significantly lower than for publicly insured patients (17% vs. 34%, p = 0.0005) (Table 4). Of those treated surgically, publicly insured patients had a greater proportion of osteotomies (81%) compared to commercially insured patients (70%). Again, no statistical analysis was performed with this sub-group analysis because of the small sample sizes.

**Table 4:** Rate of surgical treatment based on insurance type

Insurance type	Total surgery		Surgery subtypes	
	%	p-value (commercial vs. public)	Osteotomy (%)	Non-osteotomy (%)
Commercial	17% (23/138)	0.0005	70% (16/23)	30% (7/23)
Public	34% (62/183)	-	81% (50/62)	19% (12/62)
Other	50% (3/6)	-	100% (3/3)	0% (0/3)

## Discussion

The study results show that patients residing in areas with lower socioeconomic conditions (high ADI percentiles) were generally diagnosed at an older age and had higher rates of surgical treatment for DDH compared to those residing in areas with higher socioeconomic conditions (low ADI percentiles). Similarly, patients with public insurance were older at the time of diagnosis and had greater rates of surgical interventions compared to commercially insured patients. These findings clearly show the existence of disparities in DDH diagnosis timing that affect treatment protocols, especially for those in high ADI percentile areas and those with public insurance. To our knowledge, this is the first study analyzing the relationship between ADI and insurance type concerning the age at DDH diagnosis and the rate of surgical DDH treatment.

In an observational study examining data from the National Survey of Children’s Health, it was reported that Latinos, Asian/Pacific Islanders, and Native Americans faced notably higher odds of encountering challenges in accessing specialty care, particularly in non-English-speaking households [6,11]. Furthermore, Medicaid health insurance coverage was identified as a factor influencing access to pediatric specialty care [12,13].

Regarding risk factors for delayed DDH diagnosis, a study by Azzopardi et al. revealed that being born in a rural setting and having low birth weight (<2500 g) were all factors contributing to an increased likelihood of delayed DDH diagnosis occurring after three months of age [14]. Low birthweight has been strongly linked to socioeconomic inequity [15]; therefore, the association between low birthweight and late DDH diagnosis may represent a variety of undefined socioeconomic factors contributing to the delayed diagnosis. On the other hand, a retrospective cohort study conducted by Lindberg et al. showed that late presentations of DDH, defined as occurring at the age of six months or older, were more prevalent among patients with public insurance or those residing in lower-income areas [7]. Our results further delineate the socioeconomic inequities associated with delayed DDH diagnosis.

Interestingly, our results do not show a linear increase in age at DDH diagnosis or a change in treatment protocol toward surgical treatment with an increase in ADI percentile. While patients in the 1-10th ADI percentile had a younger average age at diagnosis than all other groups, the other groups did not differ significantly from each other. Additional analysis showed no significant difference in age at diagnosis or treatment protocol between patients in the 1-5th ADI percentile and those in the 6-10th ADI percentile. These results suggest that patients anywhere in the entire privileged decile of the U.S. population have an advantage over the rest of the population in terms of earlier DDH diagnosis or receiving non-operative treatment, but being closer to this top decile does not confer an increased chance of earlier diagnosis or receiving operative treatment. Given that DDH diagnosis and conservative treatment are preferred in patients before six months of age, steps to improve DDH screening and early diagnosis should be taken broadly, with the intent of enhancing the average age at diagnosis for most of the population rather than targeting a specific at-risk group. This will theoretically decrease surgery rates by lowering the average age at the detection of DDH.

Many have advocated for universal ultrasound screening for DDH, and several European countries have implemented an ultrasound screening protocol for all newborns [16]. However, not all healthcare facilities can be expected to provide ultrasound examinations for healthy newborns, especially those in underserved areas. In our perspective, optimizing the early detection of DDH could be achieved not only by depending on imaging alone but also by bolstering the training of pediatric primary care providers in the latest DDH screening methods. Additionally, enhancing parental education regarding the indicators of DDH could contribute to early identification and intervention. Gargan et al. demonstrated that the use of a simulated learning module enhances the confidence and skill level of parents learning to apply a Pavlik harness for conservative treatment of DDH [17]. Perhaps if a similar learning module were available to educate parents and caregivers on the signs of DDH and complications due to late presentation, the age at initial diagnosis could improve at our single institution. Further studies could be performed to evaluate if this would be true.

The literature is sparse regarding socioeconomic factors that influence the rate of surgical treatment for DDH. However, Murgai et al. analyzed whether Hispanic ethnicity affects surgical treatment of DDH, finding that Hispanic patients are more likely to undergo surgical treatment for DDH than non-Hispanic patients [8]. Murgai et al. also found that insurance status (public vs. private) did not impact surgical treatment rates [8]. Past studies have noted that the U.S. Hispanic population tends to have lower income and educational attainment when compared to non-Hispanic groups, particularly non-Hispanic whites [18]. While our study using ADI does not factor in ethnicity or race, the study by Murgai et al. contradicts our findings that patients with public insurance underwent a greater rate of surgery than those with commercial insurance [8]. This discrepancy may be due to the influence of confounding variables or regional differences (Los Angeles, CA, in Murgai et al. vs. Washington, DC, in our study). Further investigation is needed to understand the specific causes of increased surgical treatment rates in patients with Medicaid or other public insurance. It is also important to note that our study could not statistically analyze the effects of ADI on the type of surgical procedures performed.

Of note, the types of surgeries performed shifted more toward osteotomies for the less advantaged ADI groups as well as the public insurance group. This may be representative of more severe dysplasia, requiring a more invasive procedure as opposed to open reduction or tenotomy. The goals of screening should be to decrease surgery rates while also decreasing the need for osteotomies within those treated surgically.

This study contributes to the literature by utilizing the validated ADI measure to evaluate patients’ average age at DDH diagnosis and the rate of surgical treatment based on area deprivation status. The ADI national percentile calculation amalgamates income, education level, employment status, and housing quality at a given location to represent neighborhood disadvantage. While these variables are combined to produce one numeric value, the lack of information about how income, education, employment, and housing each contribute is a limitation. Nevertheless, the ADI score provides a convenient, holistic method to assess disadvantages, proving beneficial for this study. Other limitations of this study include its retrospective, single-center study design, which can introduce selection bias, incomplete chart data, lack of randomization, and potential confounders. Additionally, the generalizability of these findings is limited as all chart reviews were performed at a single, tertiary pediatric institution. To improve our methodology and address potential confounders, power analysis and multivariate analysis would be recommended for future studies. The number of patients in our study was restricted to those seen at our single pediatric hospital, but access to their full electronic medical records allowed us to gather home addresses for ADI and collect complete and accurate treatment data. Patients were identified through ICD-10 codes, meaning bias may be introduced due to coding errors or patients without true idiopathic etiologies. Finally, the date range that was chosen may introduce additional confounders as the true effects of the COVID-19 pandemic on DDH diagnosis and treatment have not been fully explored.

Further research is necessary to achieve a more comprehensive understanding of the limiting factors in DDH screening for patients who do not fall into the most advantaged national ADI decile. A focused, interview-based study may be beneficial to learn from parents of children diagnosed with DDH beyond six months old to understand where screening opportunities were missed or if screening efficacy could be improved. Additionally, larger cohort sizes and stratification of the study population based on the type and severity of DDH may provide further insight into how socioeconomic factors may affect the DDH population.

## Conclusions

The study results demonstrate that socioeconomic factors may play a role in the diagnosis timing and, consequently, the treatment course of DDH patients. Specifically, patients residing in areas with lower levels of deprivation tend to be diagnosed at a younger age and undergo surgical treatment less frequently. Additionally, the study highlights the need for healthcare providers and policymakers to implement targeted interventions aimed at reducing barriers to timely diagnosis and treatment, particularly for patients residing in areas with higher levels of deprivation. Fostering collaborations between healthcare institutions, community organizations, and public health agencies may prove instrumental in creating more equitable care pathways for DDH patients.
